# The burden of brucellosis in donkeys and its implications for public health and animal welfare: A systematic review and meta-analysis

**DOI:** 10.14202/vetworld.2025.367-378

**Published:** 2025-02-17

**Authors:** James Mutiiria Kithuka, Timothy Muthui Wachira, Joshua Orungo Onono, Wyckliff Ngetich

**Affiliations:** 1 Department of Public Health, Pharmacology and Toxicology, University of Nairobi, Nairobi, Kenya; 2 Department of Veterinary Surgery, Theriogenology and Medicine, Egerton University, Nakuru, Kenya

**Keywords:** brucellosis, donkeys, meta-analysis, prevalence, public health, reservoir host, systematic review, zoonosis

## Abstract

**Background and Aim:**

Brucellosis is a globally significant zoonotic disease affecting a wide range of wild and domestic animals, with implications for human and animal health. Despite donkeys’ crucial roles in agriculture, transportation, and livelihoods, there is limited research on the burden of brucellosis in this species. This study systematically reviews the prevalence and role of donkeys as reservoirs for Brucella spp., providing insights into their public health implications.

**Materials and Methods:**

Using the PRISMA guidelines, a systematic search of PubMed, Scopus, and Google Scholar was conducted for studies published from 1990 to May 2024. Out of 1159 retrieved articles, 20 met the inclusion criteria. Data on study design, location, diagnostic methods, and brucellosis prevalence were extracted and analyzed using R statistical software. Pooled prevalence and heterogeneity were calculated, and the Newcastle-Ottawa Scale was employed to assess study quality.

**Results:**

The pooled prevalence of brucellosis in 6785 donkeys across 20 studies was 10.23% (range: 0%–63.7%), with the highest prevalence reported in Asia (26.80%). While 15% of studies suggested that donkeys act as reservoirs for Brucella spp., direct evidence linking donkeys to disease transmission remains scarce. The disease’s impact on donkey reproduction, including abortion and infertility, is underexplored, highlighting a significant research gap.

**Conclusion:**

Brucellosis in donkeys represents a notable zoonotic and occupational risk. The limited data from East Africa, despite its high donkey population, emphasize the need for comprehensive epidemiological studies. Findings underscore the importance of targeted interventions, including biosecurity, public education, and enhanced diagnostic approaches, to mitigate brucellosis’ impact on donkey health and its broader public health implications.

## INTRODUCTION

Donkeys play a crucial role in agriculture, transportation, and companionship, particularly in developing regions, making their health vital for economic and social reasons [1]. Recently, the significance of milk production from species other than cattle has been highlighted to address the growing demand for milk worldwide. Donkey milk has recently gained popularity, particularly in Europe, as a substitute diet for those allergic to cows’ milk proteins and as a means of preventing metabolic diseases [2]. Donkey milk could be explored to meet this demand as well as address the issue of low animal protein (milk) intake in sub-Saharan Africa and underdeveloped countries [3, 4]. Despite playing a significant role in rural farming operations, donkeys have experienced poor treatment, lack of awareness and access to health care, and unfavorable sentiments from the local population [5]. Donkeys are vital to many subsistence strategies in semi-arid locations but compared to other domesticated species, they have received little or no attention from development agencies [6].

Despite their critical contribution to the livelihoods of donkey owners and users, the prevalence and impact of diseases in donkeys have not been thoroughly explored. Furthermore, some communities rely on milk and meat from donkeys as sources of food [7]. This calls for an examination of the potential health risks associated with these food sources to ensure that they are safe from pathogens that could spread to human beings. A few studies have reported the burden of zoonotic diseases in donkeys, including tuberculosis [8], salmonellosis [9], leptospirosis [10], anthrax [11], glanders [12], rabies [13], West Nile viral encephalitis [14], toxoplasmosis [15], hydatidosis [16], and trypanosomiasis [17].

Brucellosis, a zoonotic disease, is a potential food safety hazard posed by milk and meat. The disease is caused by a bacterium of the genus *Brucella* and is a significant public health concern globally, affecting various domestic and wild animals, including donkeys (*Equus asinus*) [18]. Although commonly associated with cattle, sheep, goats, and pigs, brucellosis in donkeys has remained poorly studied [19]. A few studies have documented the epidemiologic role of animals other than domesticated ruminants; these studies have included dogs [20], camels [21], and poultry [22]. The disease in donkeys is caused by *Brucella abortus*, *Brucella melitensis*, and *Brucella suis* [23] and is often characterized by fistulous withers, reproductive issues such as abortion, infertility, and orchitis, alongside general symptoms such as fever and lethargy [24]. The epidemiology of this disease in donkeys is not completely known. The donkeys may contract the disease by grazing near affected animals, contaminated dust or droplets, or watering spots [23]. Brucellosis in donkeys can be diagnosed using a range of serological and immunological tests. Commonly employed methods include agglutination-based tests such as the Rose Bengal plate test (RBPT), tube agglutination test (TAT), microtiter serum agglutination test, serum agglutination test, serum plate agglutination test (SPAT), and buffered acidified plate antigen test [18–20, 24]. These tests are primarily used for initial screening. The standard tube agglutination (STAT) test provides a more standardized approach to confirmation. Advanced serological assays, including competitive enzyme-linked immunosorbent assay (cELISA) and indirect enzyme-linked immunosorbent assay, have higher sensitivity and specificity [24]. In addition, the complement fixation test (CMT) is used for confirmatory diagnosis and to meet international standards in brucellosis testing. These diverse methods enable comprehensive screening and accurate diagnosis of brucellosis in donkeys.

The implications of brucellosis in donkeys are multifaceted, affecting the well-being and productivity of infected donkeys and other animals and posing serious zoonotic risks to humans. With the several welfare-related challenges facing donkeys, diseases such as brucellosis can intensify the already worse welfare status of working donkeys [5]. It has also been argued that donkeys may act as a reservoir for brucellosis [23]. Given the close interaction between donkey users and owners, especially in rural areas, the zoonotic potential of brucellosis calls for a comprehensive understanding of its prevalence, transmission, risk factors, and control measures in these animals. Unlike in donkeys, brucellosis has been extensively studied in horses from different countries with significantly varied prevalence; 60.59% in Van province of Turkey [25], 0.24% in Mexico [26], 9.5% in Hakkari-Turkey [27], 2.5% in Mashhad-Iran [28], 20.7% in Faisalabad-Pakistan [29], 2.5% in Northeast of Iran [30], 14.7% in North Nigeria [31], and 13.68% in Southeast Turkey [32].

This study aimed to address three critical research questions related to brucellosis in donkeys. First, this study aimed to estimate the prevalence of brucellosis in donkeys and how this compares in different regions and countries. This comparison will provide insights into the relative risk and exposure levels in different countries. Second, the role of donkeys as reservoir hosts for brucellosis and their contribution to the transmission dynamics of the disease to humans and other animals are examined. Understanding these dynamics is essential for developing effective control and prevention strategies. Third, the burden of brucellosis on donkey reproduction rates, alongside its effects on breeding programs and overall donkey populations. The findings will inform future research directions and policy decisions, ultimately contributing to better management and control of brucellosis in donkeys and reducing the risk of occupational exposure for those in contact with donkeys and their products.

## MATERIALS AND METHODS

### Ethical approval

To carry out this systematic review, we used the Preferred Reporting Items for Systematic Reviews and Meta-Analyses checklist and guidelines [33]. The protocol was prepared before study commencement and can be obtained from the corresponding author upon request.

### Study period and location

The literature search, data collection, and data analysis were conducted at the Faculty of Veterinary Medicine, University of Nairobi, from April 2024 to September 2024. The included studies were published between 1990 and May 2024. The included studies were conducted in 10 countries: Africa, Asia, Europe, and South America.

### Search strategy

Using the publish or perish platform [34], we used the following databases to search for relevant studies – PubMed, Scopus, and Google Scholar. For each research question, search terms were generated to identify as many articles as possible. For the first question, the following terms were used “prevalence of brucellosis in donkeys,” “donkey brucellosis epidemiology,” “brucellosis in donkeys in different countries,” and “brucellosis infection rates in donkeys” and for the second question, “donkeys as brucellosis reservoir hosts,” “brucellosis transmission dynamics in donkeys,” “donkeys’ brucellosis zoonotic potential,” “brucellosis transmission from donkeys to humans,” “animal reservoirs of brucellosis,” and “brucellosis in donkeys and disease spread.” Boolean operators “AND” and “OR” were used to combine the terms during the search. To identify original papers that might have been missed during the search process, we manually searched for further relevant research using references from the retrieved articles and associated systematic reviews. The Rayyan platform [35] was used to remove duplicate articles and organize search outcomes for either relevant or irrelevant studies based on the inclusion/exclusion criteria.

### Inclusion and exclusion criteria

#### Research questions

(i) What is the prevalence of brucellosis in donkeys and how is it compared between males and females?
(ii) What is the role of donkeys as reservoir hosts for brucellosis and how does this contribute to the transmission dynamics of the disease to humans and other animals?

#### Inclusion criteria

Observational studies with the prevalence/incidence of brucellosis in donkeys as the outcome or reported donkeys as potential reservoirs for brucellosis written in English. There was no limitation on the region of the studies, but records published between 1990 and May 2024 were included in the study.

#### Exclusion criteria

Case reports, case series, review articles, experimental studies, studies not reporting prevalence/incidence as the outcome, and unpublished/gray literature were excluded from the study.

### Selection of articles

This study used the patient/population, intervention, comparison, and outcome approach to identify the relevant articles where Population (P): Donkeys, Exposure (E): Diagnostic/screening tests such as serology and molecular to detect brucellosis, Comparison (C): Not applicable, and Outcome (O): Prevalence/incidence of donkey brucellosis and reported donkeys as potential reservoirs. Two researchers independently screened the articles, and a third researcher was consulted to settle any discrepancies. Two phases were used to select the articles, whereas phase one involved reviewing the title and abstract of the articles and categorizing them based on the inclusion/exclusion criteria as either included, excluded, or maybe. Articles that were unclear as to be included or not were downloaded for further screening and then classified accordingly. In the second phase, all relevant articles were downloaded, and a detailed review of the full text was performed to select appropriate articles.

### Data extraction

Using the designed data extraction tool, data were extracted from the appropriate articles, including the following: Name of the first author, year of publication, study design, continent, country, laboratory method of identification, total sample size, sex distribution, overall prevalence of brucellosis, and prevalence in different sexes where applicable.

### Data quality assessment

The Newcastle–Ottawa Scale (NOS) [36] was modified by adding more parameters for each domain as described below and used to assess the quality of the data collected based on three broad criteria/domains: (1) Selection bias; this domain was assessed by looking at the study during the selection of donkeys if the following aspects were considered – randomization, inclusion/exclusion criteria, stratified sampling (subgroups, e.g., sex, location, and production systems) and representativeness (sample size >20). This domain receives a maximum of four points if all the issues are captured. (2) Ascertainment of outcome: This assesses the test used to generate results, the data analysis performed to determine if it is credible, and the selective reporting (only reporting the positive). This domain receives a maximum of three points. (3) Reporting and transparency: this domain assessed the detailed description of the study design, sampling methods, recruitment strategies, and data collection procedures. Similarly, if the articles transparently reported, any limitations related to selection bias and discussed their potential impact on the study findings. This domain receives four points at maximum. The maximum quality score was 11 points for each article. We regarded publications with a total score of 8–11 points to be of high quality, whereas 4–7 points represented moderate quality and scores of 0–3 represented low quality.

Each included study was assessed and scored, and the scores were used to summarize the quality of the evidence, identify high-quality studies, and weigh the evidence in the systematic review. The domain-specific scores highlight areas where studies may have potential biases, such as selection or comparability, thereby aiding in the critical appraisal of the evidence. The NOS is a robust and practical tool that provides a standardized method for assessing the quality and risk of bias in observational studies, facilitating the synthesis of reliable and valid findings in systematic reviews.

### Statistical analysis

Data variables collected in Excel, 2021 (Microsoft Corporation, Washington, USA) were exported to R statistical software version R 4.1.2 for statistical analysis using the meta and metafor packages. Frequencies, summaries, and proportions were calculated and presented as tables and figures. Pooled prevalence was determined using a random-effects model to account for between-study variability. Confidence intervals (95%) for pooled estimates were computed to provide a measure of uncertainty.

Heterogeneity across studies was assessed using Cochran’s Q test and quantified with the I² statistic, which categorizes heterogeneity as low (I² = 25%), moderate (I² = 50%), or high (I² = 75%). A significance threshold of p < 0.10 was applied for the Q test, given its lower statistical power in meta-analyses with few studies. Potential sources of heterogeneity, such as diagnostic tests, geographic regions, and sample characteristics, were explored using subgroup analyses and meta-regression models. Predictor variables were selected based on theoretical relevance and data availability, and their influence was assessed at p < 0.05.

Forest plots were generated to visualize individual study estimates and pooled prevalence, with confidence intervals for each effect size. Funnel plots were created to evaluate publication bias, complemented by Egger’s regression test to statistically detect asymmetry (p < 0.10). Sensitivity analyses were conducted by removing one study at a time (leave-one-out analysis) to assess the stability and robustness of the pooled estimates. Diagnostic tests were compared using pairwise comparisons and meta-regression to evaluate differences in sensitivity and specificity. Data were stratified for studies using multiple diagnostic methods to account for test-specific performance. To ensure robustness, all analyses were repeated under both fixed-effects and random-effects models, with results compared for consistency.

All statistical analyses were conducted with strict adherence to reporting standards and ensuring reproducibility and transparency.

## RESULTS

**Figure 1:**
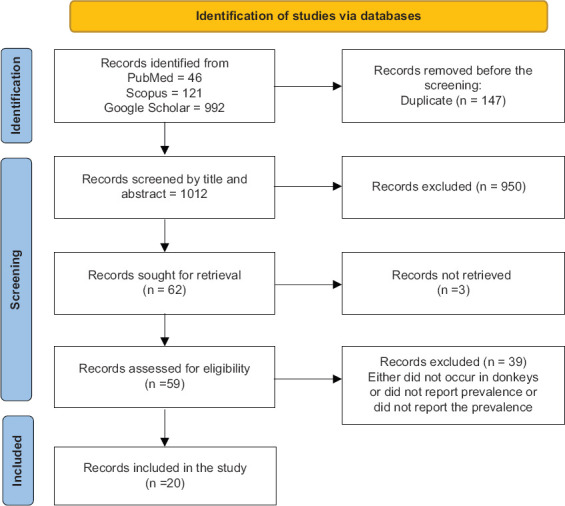
Flow diagram of the record selection process.

Out of 1159 articles identified from the selected databases based on inclusion and exclusion criteria, 46 were retrieved from PubMed, 121 from Scopus, and 992 from Google Scholar. Of these, 147 duplicate studies were excluded. After screening the title and abstracts, 950 records were excluded from not meeting the inclusion requirements; either they were irrelevant or missing. The full texts of the 62 remaining articles were downloaded and assessed, and 42 were excluded, including three articles with no available full texts or that were not accessible, and 39 articles that were either conducted on other equids (horses and mules) or did not report the prevalence of brucellosis. Ultimately, 20 articles were included in this study (Figure 1 and Table 1 [18,24,26,29,32,37–50]).

**Table 1:** Characteristics of eligible studies and reported prevalence of donkey brucellosis from different countries.

Country	Sample size	Males	Females	Laboratory test	Overall prevalence	Infected males (%)	Infected females (%)	References
Nigeria	200	105	95	RBPT	21.5	23 (22)	19 (20)	[18]
Nigeria		105	95	iELISA	18.5	20 (19)	17 (17.9)	[18]
Brazil	178	N/A	N/A	RBPT&STAT	0	N/A	N/A	[24]
Mexico	86	74	12	RBPT	0	N/A	N/A	[26]
Pakistan	160	74	86	RBPT	4.4	N/A	N/A	[29]
Pakistan		74	86	SAT	3.8	1 (1.4)	3 (3.4)	[29]
Turkey	1172	N/A	N/A	RBPT	6.1	N/A	N/A	[32]
Turkey		N/A	N/A	SAT	0.5	N/A	N/A	[32]
Nigeria	300	200	100	RBPT&MSAT	5	3 (1.7)	1 (1.3)	[37]
Nigeria		200	100	cELISA	3.3	3 (1.3)	2 (2)	[38]
Nigeria	600	393	207	RBPT&MSAT	5.5	9 (2.3)	6 (2.9)	[38]
Nigeria	601	374	227	RBPT	7.2	33 (8.8)	10 (4.4)	[39]
Nigeria		374	227	cELISA	6.7	32 (8.6)	8 (3.5)	[39]
Nigeria	1000	585	415	RBPT&MSAT	11.4	51 (8.7)	63 (15.2)	[40]
Pakistan	267	N/A	N/A	SPAT	63.7	(41.7)	(58.2)	[41]
Pakistan	8	N/A	N/A	RBPT&iELISA	0	N/A	N/A	[42]
Sudan	28	N/A	N/A	RBPT&SAT&CMT	3.6	N/A	N/A	[43]
Sudan	412	N/A	N/A	RBPT	2.1	N/A	N/A	[44]
Sudan	150	143	7	RBPT	24	36 (25.1)	0	[45]
Brazil	110	N/A	N/A	RBPT	0.9	N/A	N/A	[46]
Egypt	423	150	273	RBPT	1.7	1 (0.7)	6 (2.2)	[47]
Egypt		150	273	BAPAT	2.2	1 (0.7)	8 (2.9)	[47]
Egypt		150	273	TAT	1.4	1 (0.7)	5 (1.8)	[47]
Algeria	120	115	124	RBPT	0	N/A	N/A	[48]
Jordan	120	58	62	RBPT	8.5	N/A	N/A	[49]
India	166	N/A	N/A	TAT	3.6	N/A	N/A	[50]

Some studies used more than one laboratory test and therefore appeared more than once, and some studies did not provide details on the sex distribution of the included donkeys. RBPT=Rose Bengal plate test, TAT=Tube agglutination test, MSAT=Microtiter serum agglutination test, SAT=Serum agglutination test, SPAT=Serum plate agglutination test, BAPAT=Buffered acidified plate antigen test, STAT=standard tube agglutination, cELISA=competitive enzyme-linked immunosorbent assay, iELISA=Indirect enzyme-linked immunosorbent assay, CMT=complement fixation test

Out of these, only three articles reported the use of donkeys as a potential reservoir for brucellosis. However, the third question was excluded because no explicit data were available that directly reported the effect of brucellosis on the reproduction and breeding of donkeys.

### Pooled prevalence estimates of brucellosis in donkeys and heterogeneity

**Figure 2:**
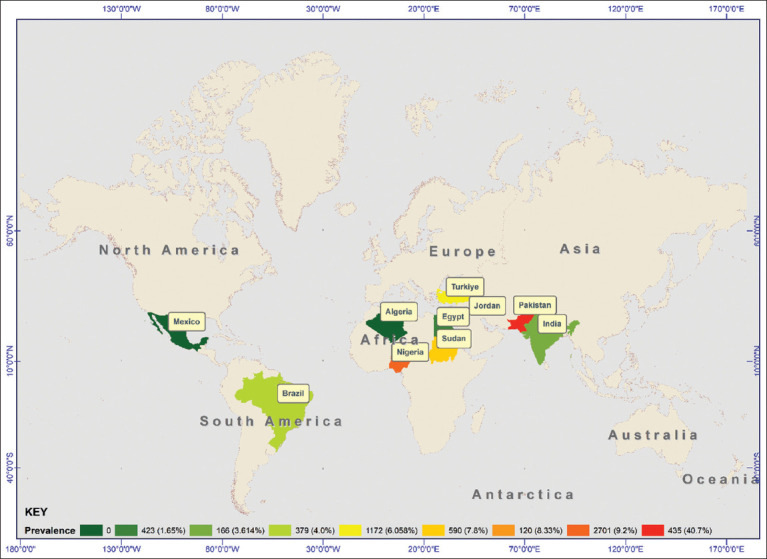
Distribution of the prevalence of donkey brucellosis in different countries according to the reviewed articles. Most articles are from Africa, mainly from the western and northern parts, and a few from different continents [Source: The map was generated using ArcMap version 10.8.2., ESRI, California, USA).

**Figure 3:**
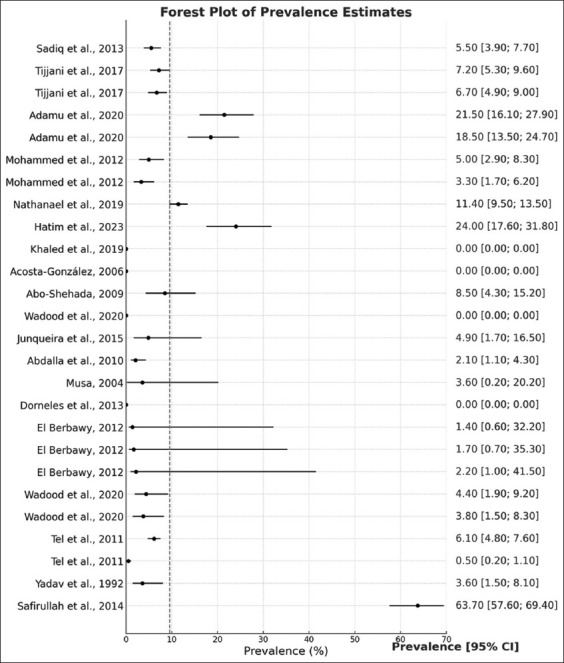
Forest plot shows reported prevalence and 95% confidence intervals of brucellosis in donkeys from eligible studies. Substantial statistical heterogeneity was estimated from the effect size of each individual study.

From the reviewed articles, a total of 6,785 donkeys were included, of which 694 tested positive for brucellosis, yielding a pooled prevalence of 10.23%. The reported prevalence ranged from 0% to 63.7%. Across different continents, the highest pooled prevalence was observed in Asia (26.8%), whereas no donkeys in North America were found to have antibodies against the *Brucella* bacterium. From each country, a higher (40.7%) pooled prevalence was reported in Pakistan, followed by 9.2%, 7.8%, and 4.0% from Nigeria, Sudan, and Brazil, respectively (Figure 2). However, other countries had only a single article, and pooled prevalence was not estimated (Table 2). The individual study prevalence effect size yielded substantial statistical heterogeneity (I^2^ = 97%, χ^2^ = 181.6437, p < 0.01) (Figure 3).

**Table 2:** Pooled prevalence estimates from eligible articles from different countries.

Country	Samples tested	Samples positive	Pooled prevalence (CI: 95%)	Prevalence range (%)
Nigeria	2701	248	9.2 (8.1–10.3)	3.3–21.5
Pakistan	435	177	40.7 (36.1–45.5)	0.0–63.7
Sudan	590	46	7.8 (5.8–10.3)	2.1–24
Brazil	379	15	4.0 (2.3–6.6)	0.0–5.5
Egypt	423	7		1.7
Turkey	1172	71		6.1
Algeria	120	0		0.0
Mexico	86	0		0.0
Jordan	120	10		8.5
India	166	6		3.6

CI=Confidence interval

### Eligible articles

Of the 20 articles included in this study, most were from Africa (50%; 10/20), followed by Asia (25%; 5/20), South America (15%; 3/20), and 5% (1/20), each from Europe and North America continents.

**Figure 4:**
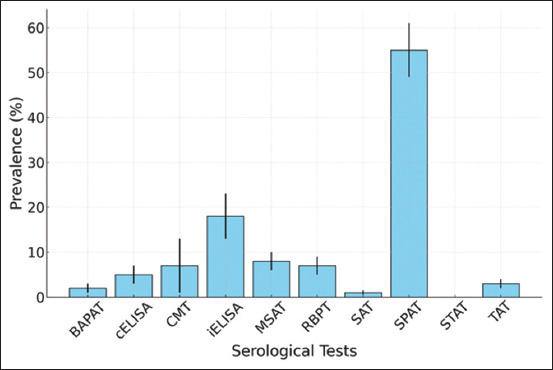
Comparison of prevalence according to serological test results.

For screening, the RBPT was used, and the positive samples were subjected to either an agglutination test (72.7%; 8/11), which is considered a gold standard test, ELISA, either indirect or competitive (18.2%; 2/11), or CMT (9.1%; 1/11). However, the other studies used only one diagnostic test: RBPT (77.8%; 7/9), TAT (11.1%; 1/9), or SPAT (11.1%; 1/9) (Table 3 and Figure 4). There was a significant heterogeneity (I^2^=98.8%) across the different serological tests (χ^2^ = 1312.4, df = 9, p < 2.2e-16).

**Table 3:** Pooled prevalence of donkey brucellosis according to laboratory test results.

Test	Samples tested	Samples positive	Pooled prevalence (CI: 95%)	p-value	I² (Common effect)	I^2^ (Random effect)
RBPT	6182	512	8.3 (7.6–9.0)	Reference	51.5	10.2
TAT	589	12	2.0 (1.1–3.6)	<0.01	4.9	10.1
MSAT	1900	162	8.5 (7.3–9.9)	0.77	15.8	10.2
SAT	1332	12	0.9 (0.4–1.6)	<0.01	11.1	10.2
SPAT	267	170	63.8 (57.6–69.4)	<0.01	2.2	10.1
iELISA	200	37	18.5 (13.5–24.7)	<0.01	1.7	10.0
cELISA	901	50	5.5 (4.2–7.3)	0.006	7.5	10.2
STAT	178	0	0 (0)	0.01	1.5	10.0
BAPAT	423	9	2.1 (1.0–4.1)	<0.01	3.5	10.1
CMT	28	1	3.6 (0.2–20.2)	0.57	0.2	9.0

CI=Confidence interval, RBPT=Rose Bengal plate test, TAT=Tube agglutination test, MSAT=Microtiter serum agglutination test, SAT=Serum agglutination test, SPAT=Serum plate agglutination test, BAPAT=Buffered acidified plate antigen test, STAT=Standard tube agglutination, cELISA=competitive enzyme-linked immunosorbent assay, iELISA=indirect enzyme-linked immunosorbent assay, CMT=Complement fixation test

Of these articles, only nine reported the prevalence of brucellosis disaggregated by sex, with males ranging from 0.7% to 41.7% and females from 0% to 58.2%. Sex was reported to be significantly associated with brucellosis seroprevalence in 55.6% (5/9) of these articles, and in four of them, females had higher odds (1.5–3) of testing positive compared to males, with only one reporting males having a higher risk of infection. It was not possible to estimate the pooled prevalence and effect of associations for males and females separately since most of the reports did not provide details on the distribution of donkeys by sex. However, other factors such as age, herd size, management practices, and donkey use were reported as potential risk factors for brucellosis.

### Donkeys as potential reservoirs for brucellosis

Only 3 (15%) of the included articles reported donkeys as potential reservoirs of brucellosis and act as a source of infection to other animals, including humans [24, 38, 18]. These reports only implicated the donkey as a potential reservoir, but there was no direct link. The serological tests are used to detect antibodies that suggest exposure and not active infection. These reports by Ocholi *et al*. [51] are mostly extrapolations of similar findings from other equines, especially horses, which spread the disease to other animals. For example, cattle and dogs are reported to contract brucellosis from infected mares [24]. Akinyemi *et al*. [52] have reported brucellosis in donkeys on farms and previously reported the disease in cattle, but the infection’s origin was unclear. The chronic nature of brucellosis and fistulous withers in equines ensures continuous discharge and contamination of feed, water, and objects that can spread the disease to other animals.

## DISCUSSION

This review shows that there are limited studies on brucellosis in donkeys, despite their immense contribution to the lives and livelihoods of rural populations. Donkeys in East Africa play a critical role in poverty reduction, and the current increase in the use of donkey milk places humans at risk of contracting zoonotic diseases. Several zoonoses in equines, including brucellosis, have been reviewed [52, 53], but little attention has been paid to the potential risk posed by the close contact between people and donkeys. Donkeys graze closer to the soil than other livestock, so they are more likely to pick pathogens. With the nature of their work as transport animals, they are likely to move to various regions and interact with other animals, increasing the risk of disease spread. With increasing awareness of animal welfare, there is currently a surge in studies on donkey well-being and welfare, but few studies have examined the potential public health risks of donkey diseases. Brucellosis is an important disease in donkeys because of its impact on work capacity and reproduction and because infected donkeys could be a potential source of infection to other animals, including humans [24]. Brucellosis in donkeys is caused by *B. abortus*, *B. melitensis*, and *B. suis;* however, molecular analysis is needed to determine the actual species and strains. Individuals who work in close contact with donkeys, such as farmers, slaughterhouse employees, hunters, and veterinarians, are considered high-risk groups [54]. Laine *et al*. [55] have suggested that the human incidence of brucellosis reflects the true epidemiological situation of brucellosis in the animal population. With climate change, the geographic distribution of brucellosis is continually shifting, with new foci emerging or re-emerging [56]. Animal-to-human transmission of brucellosis is primarily determined by the animal reservoir as well as several other variables, such as consuming raw or inadequately heat-treated milk and milk products, human contact with animals without following biosafety protocols, including handling and manipulating viscera and handling animal fluids without wearing personal protection equipment, as well as climatic circumstances [57]. Serological tests are typically used to diagnose brucellosis because the culture of *Brucella* spp. is extremely risky and requires a laboratory with a high level of biosecurity [58].

This review established that there is scarce literature on donkey brucellosis, as most studies were carried out following the increasing burden of the disease in other livestock, such as cattle, sheep, and goats [39]. A higher pooled prevalence of 26.8% was reported in Asia, but this can be because of the small sample of donkeys compared with those from Africa. The wide range (0%–63.7%) of reported prevalence from different studies can be attributed to differences in serological assays, cutoff points used, sample size, and representativeness of the sampling. From the studies reviewed, more male donkeys were sampled than females, which may have been attributed to most donkey owners and users preferring male donkeys, as females are considered not strong and would cease working when pregnant [45]. Similar findings were reported in a donkey welfare study in Kenya, where 93.7% of the assessed donkeys were males [5]. In studies of brucellosis in other animal species, similar findings showed that bulls had greater seroprevalence rates than cows [59], and stallions had higher seroprevalence rates than mares [18].

Brucellosis has been researched extensively in other animal species, and various findings have been reported. For instance, in Kenya, a seroprevalence of 22.9% was reported in cattle, 20% in camels, 15.5% in goats, and 8.6% in sheep from Baringo County, Kenya [60]. Elsewhere, brucellosis has been reported by Akinyemi *et al*. [52] in cattle from different countries, accounting for 13.3% in Nigeria, 4% in Ethiopia [61], and 1.1% in Eritrea [62].

In cattle, brucellosis has been reported to cause a 20%–25% reduction in milk production, 10%–15% in meat production, and a 15% loss of calves owing to abortions, a 30% increase in the rate of animal replacement, and a prolonged calving interval of up to 11.5–20 months [63]. It has also been reported that one in every five infected animals either aborts or becomes permanently infertile. Brucellosis is a notifiable disease in most countries, but because of its vague clinical manifestations, it is usually underreported, and the few reports constitute a small fraction of the true burden of the disease.

Previous studies have implicated the donkey as a potential reservoir of *Brucella* bacterium [18, 31] and as a source of infection to other animals, including humans [24, 51]. The close interaction between donkeys and humans increases the risk of exposure to zoonotic diseases, including brucellosis [18]. However, there was no direct link between donkeys and the spread of the disease to other animals. Because of the risks associated with the isolation of bacteria, serological tests are commonly used to determine the burden of the disease; however, these are mainly used to assess exposure and may not indicate actual infection. However, other species have been reported as potential sources of infection in equines and vice versa including dogs and domestic ruminants [24]. It is believed that bacteria reach the equines through contact with aborted material and vaginal secretions from pigs and cattle [24]. However, other studies have argued that despite donkeys and bitches being potential sources of infection, they are not significant for spreading the disease to other animals [42]. Moreover, Wisniewski and Ranus [64] reported that two cows and a dog contracted brucellosis from a mare with recurring disease abscesses. However, other investigations by Junqueira *et al*. [46] have shown that mares with infectious discharge during the postpartum or post-abortion period did not spread the disease to other horses or cows. Similarly, when the experimentally infected mare was allowed to interact with the heifers, *Brucella* was not isolated from the heifers [65]. However, there is evidence that wild mammals [66], such as white-tailed deer, opossums, raccoons [67], hares [68], and pampas deer (*Ozotocerus bezoarticus*) [69] do act as reservoirs of *B. abortus* based on epidemiologic research that examined the involvement of various species as reservoirs. Donkeys have been implicated as reservoirs for brucella, but there is scarce literature on this issue. Therefore, donkeys need to be included in epidemiological studies on brucellosis in livestock. Similarly, this calls for phylogenetic analysis of the isolates from livestock to check for genetic relatedness and genetic evolution of the *Brucella* serovars.

Because of the few studies on brucellosis in donkeys and the non-specific signs, infection might be circulating in the donkey population without detection [18, 24, 60, 62, 65]. In other animal species, classical signs are usually associated with the skeletal system, such as bursitis, synovitis, osteoarthritis, and osteomyelitis, and reproductive system, such as orchitis and epididymitis in males and abortions in females [70]. These signs are usually inapparent, and given the limited attention given to donkey health, diagnosis in donkeys may be difficult. Infected donkeys, however, can experience infertility or reduced fertility in both males and females [18]. The disease can cause orchitis and epididymitis in males, leading to decreased sperm quality and viability and infection transmission through breeding. In females, brucellosis often results in placentitis, which compromises fetal development and increases the likelihood of abortion and subsequent infertility [24]. Chronic infections may cause persistent reproductive failure [18], which can significantly impact efforts to improve the breeding technology of donkeys. Similarly, chronic brucellosis in other equines is associated with weight loss, lameness, and general debilitation, impairing their ability to work and reducing their overall productivity [71]. The presence of *Brucella* in the reproductive organs and tissues of infected donkeys can also facilitate the spread of the disease within herds, posing a significant challenge in breeding programs [72].

None of the studies reviewed here have associated seropositivity with any reproductive parameter, as the positive samples were from clinically healthy donkeys. Similarly, with limited studies on donkey reproduction and breeding, there is scarce literature on the impact of brucellosis on the reproductive performance of donkeys. However, the disease has been studied more in horses, which may be attributed to the higher percentage of horses than donkeys. In horses, it has been reported widely; 60.59% in Turkey [25], 0.24% in Mexico [26], 9.5% in Hakkari-Turkey [27], 2.5% in Mashhad-Iran [28], 2.5% in Northeast of Iran [30], 14.7% in North Nigeria [31], and 13.68% in Southeast Turkey [32], 0.0% in Eritrea [62], 20.7% in Faisalabad-Pakistan [73]. With the increasing demand for donkey skin, donkey theft, and illegal slaughter that have been witnessed globally in the last decade, there is potential for zoonotic diseases like brucellosis to spread [74]. Hence, the donkey population will be substantially reduced [75]. With the slow reproductive performance and lack of proper breeding programs for donkeys, their population is declining, and this is the time to take action. There is a need to research the reproductive health of donkeys and push for the adoption of new technologies to enhance reproductive performance to ensure that the donkey population increases [18].

## CONCLUSION

This study highlights the global prevalence of brucellosis in donkeys, with a pooled prevalence of 10.23% based on 20 eligible studies involving 6785 donkeys. The highest prevalence was observed in Asia (26.8%), while no cases were reported in North America. The findings underscore the role of donkeys as potential reservoirs of Brucella spp., with implications for both public health and animal welfare. However, direct evidence of transmission from donkeys to other species remains limited.

The strength of this study lies in its comprehensive approach, utilizing systematic review and meta-analysis to provide a global perspective on the burden of brucellosis in donkeys. The inclusion of diverse geographic regions and diagnostic methods adds robustness to the findings. In addition, the use of advanced statistical techniques, such as subgroup analysis and meta-regression, has enabled the identification of heterogeneity sources and provided deeper insights into the prevalence variations across regions and diagnostic tests.

Despite these strengths, the study faced limitations, including a reliance on secondary data, potential publication bias, and heterogeneity across studies in terms of diagnostic methods and sample characteristics. The absence of data from some regions, such as East Africa, and the lack of studies on the impact of brucellosis on donkey reproduction highlight significant research gaps.

Future research should prioritize epidemiological studies in underrepresented regions, particularly East Africa, to better understand the prevalence and transmission dynamics of brucellosis in donkeys. In addition, there is a need for studies focusing on the reproductive health impacts of brucellosis in donkeys, which remain poorly explored. Molecular studies to identify specific Brucella strains and their zoonotic potential would further enhance understanding and inform targeted interventions.

This study provides a critical foundation for improving the management and control of brucellosis in donkeys. By addressing the identified research gaps, future efforts can contribute to enhanced public health, animal welfare, and the sustainable use of donkeys in various socioeconomic roles.

## AUTHORS’ CONTRIBUTIONS

JMK: Conceptualization, design, and writing–original draft. TMW, JOO, and WN: Conceptualized and designed the study, interpreted the results, and reviewed and edited the manuscript. JMK and WN: Articles screening, data extraction, data analysis, visualization, and interpretation of results. All authors have read and approved the final manuscript.
